# Discharge teaching, patient-reported discharge readiness and postsurgical outcomes in gynecologic patients undergoing day surgery: a generalized estimating equation

**DOI:** 10.1186/s12893-022-01607-x

**Published:** 2022-05-10

**Authors:** Huaxuan You, Anjiang Lei, Xin Li, Xu Liao, Jing Chang

**Affiliations:** 1 Department of Obstetrics and Gynecology Nursing, West China Second University Hospital, Sichuan University/West China School of Nursing, Sichuan University, Chengdu, China; 2 Key Laboratory of Birth Defects and Related Diseases of Women and Children (Sichuan University), Ministry of Education, Chengdu, Sichuan China

**Keywords:** Discharge teaching, Discharge readiness, Postsurgical outcomes, Day surgery, Gynecological patients

## Abstract

**Background:**

Gynecologic patients undergoing day surgery are discharged in an intermediate stage of recovery. The quality of discharge teaching and discharge readiness are important to patients’ postsurgical outcomes, but little research has focused on them.

**Methods:**

Quality of discharge teaching and discharge readiness were measured, and Spearman correlations were conducted. Postsurgical outcomes were recorded on postoperative Day 1, postoperative Day 7, and postoperative Day 28. Generalized estimating equations were used to explore factors that influence postsurgical outcomes.

**Results:**

Discharge teaching was verified to be positively correlated with the discharge readiness of participants. The generalized estimating equations indicated that discharge teaching skills, effects of doctors and nurses, patient-reported physical conditions and social support following discharge were protective factors for postsurgical outcomes.

**Conclusions:**

Doctors and nurses should improve discharge teaching skills and effects to improve the postsurgical outcomes of gynecological patients undergoing day surgery. At discharge, doctors and nurses should assess patients’ physical condition and facilitate a social support system.

## Background

Gynecological disorders are diseases of the female reproductive system, ranging from endocrine disorders and infection to various benign and malignant tumors [1, 2]. Gynecological disorders often cause abnormal uterine bleeding, pelvic pain, chronic anovulation, infertility, and even death, impacting women’s quality of life and mental health [3–5]. Surgery is one of the main treatments for most gynecological diseases. With the widespread use of minimally invasive technology in the surgical field, minimally invasive gynecologic surgery now plays an integral part in the treatment of benign and malignant gynecologic conditions [6, 7]. Compared with laparotomy, the advantages of minimally invasive gynecologic surgery include smaller incisions, less pain, fewer complications, faster recovery and shorter hospital stays [7, 8].

Concurrent with the rise of minimally invasive gynecologic surgery, the number of day gynecological surgeries has increased considerably [9, 10]. The advantages of day surgery include convenient scheduling, cost savings and higher patient satisfaction [11, 12]. Minimizing the length of hospital stays means that patients are discharged in an intermediate stage of recovery. A shorter length of hospital stay has been positively associated with postdischarge emergency visits [13]. Therefore, discharge education about postoperative self-care and strategies to manage postdischarge problems is crucial because these provisions can enable patients to recognize when medical intervention is required [14]. However, the decreased length of hospital stay reduces the time available for doctors and nurses to provide health education, which might cause discharge teaching to be delivered in a rush [15]. As reported, improved discharge teaching was related to hospital readmission [16, 17]. Therefore, the quality of discharge teaching for patients who undergo day gynecologic surgery should be investigated, but little research has focused on it.

Readiness for hospital discharge has been described as an estimate of the ability of patients and family members to leave the hospital. Readiness for hospital discharge is the perception of being prepared or not for hospital discharge [18, 19]. Qualified readiness for hospital discharge indicates that the patients have sufficiently recovered clinically to be safely discharged [20]. Generally, readiness for hospital discharge is a medical decision based on the achievement of clinical criteria. The patients’ perception of readiness for hospital discharge may be different from their caregivers’ assessments [19]. Patient-reported discharge readiness has been posited as a potential predictor of postdischarge outcomes [21]. Moreover, readiness for hospital discharge was verified to be related to quality of discharge teaching and hospital readmission, and readiness for hospital discharge played an intermediary role in the interaction mechanism of the three variables [22–24]. Previous studies investigated the readiness for hospital discharge for parents of hospitalized children, postpartum women, general surgical patients and cataract patients [14, 22, 23, 25], but little research focused on patients who underwent day gynecologic surgery. Minimizing the length of hospital stay for patients undergoing day gynecological surgery may have an impact on their readiness for hospital discharge, which must be investigated.

As mentioned above, postdischarge outcomes were reported to be related to the quality of discharge teaching and readiness for hospital discharge among children, postpartum women, general surgical patients and cataract patients [14, 22, 23, 25]. For patients undergoing day gynecological surgery, the postsurgical outcomes were used to evaluate the postdischarge health outcomes. At present, the correlations between discharge teaching, patient-reported discharge readiness and postsurgical outcomes in patients undergoing day gynecologic surgery are still unclear. To verify the correlations between the three variables, three telephone interviews were conducted with patients to investigate these postsurgical outcomes. Most patient-reported problems after day gynecologic surgery include constipation, diarrhea, pain, vaginal bleeding, urinary tract infection and surgical site infection [26–28]. In this study, we included these problems on our postsurgical outcomes form.

**Fig. 1:**
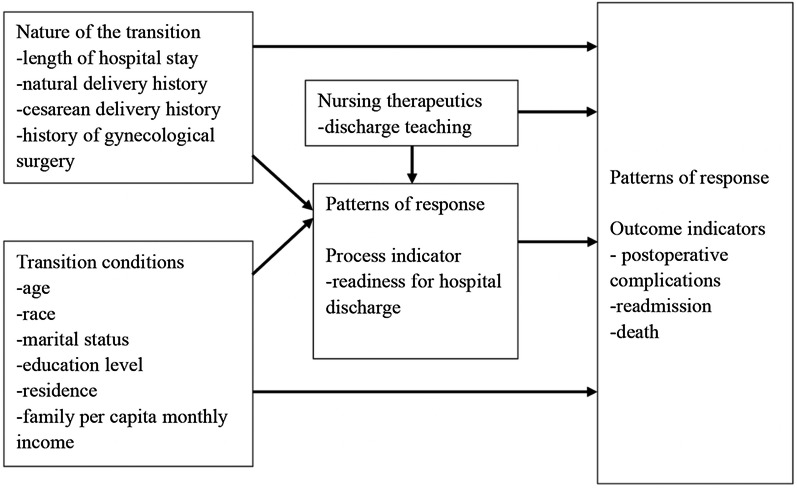
The theoretical framework based on Meleis’ middle-range theory of transitions

Meleis’ middle-range theory of transitions was selected as the theoretical framework for this study, which is widely used in transitional care studies [29–31]. Hospital discharge was regarded as a transitional process. The congruence between the concepts of the middle-range theory of transitions and the transition of postdischarge patients conceptualized the discharge transition and identified relevant study variables [18]. Four main dimensions of the middle-range theory of transitions were applied in this study: (1) nature of the transition (length of hospital stay), (2) transition conditions (patient characteristics), (3) medical therapeutics (discharge teaching), and (4) patterns of response (readiness for hospital discharge, postsurgical outcomes) [18, 22, 23]. The process indicator was readiness for hospital discharge, and the outcome indicators were postsurgical outcomes, which was the dependent variable in this study. Figure 1 depicts the theoretical framework.

The aims of this study are (1) to investigate the level of discharge teaching and readiness for hospital discharge of patients undergoing day gynecologic surgery; (2) to explore the correlation between discharge teaching and readiness for hospital discharge of patients undergoing day gynecologic surgery; and (3) to explore the influencing factors of postsurgical outcomes for patients undergoing day gynecologic surgery. Two hypotheses based on the middle-range theory of transitions are proposed: (1) Discharge teaching is correlated with readiness for hospital discharge for patients undergoing day gynecologic surgery; (2) Discharge teaching and readiness for hospital discharge are the influencing factors of postsurgical outcomes for patients undergoing day gynecologic surgery.

## Design and methods

This was a STROBE checklist-compliant longitudinal study.

### Setting and participants

We conducted a longitudinal survey from January 2021 to June 2021 in a women and children’s medical center in western China serving over 5 provinces. Convenience sampling was used. According to Kendall’s experience and methods, the sample size can be 5 to 10 times the number of independent variables [32]. Our study examined a total of 19 independent variables (9 social demographic data points, 5 questions from the Quality of Discharge Teaching Scale, 5 from the Readiness for Hospital Discharge Scale). Our sample size was calculated as 85 to 190.

Patients were eligible if they (1) were diagnosed with gynecological disease, (2) were underwent day surgery, (3) were over 18 years old, (4) were able to read and write Chinese, and (5) had normal cognitive function and intelligence. Patients were excluded if they (1) had gynecologic cancer, (2) had other severe organic diseases, or (3) had a history of mental illness.

### Data collection

A self-designed questionnaire was used to collect sociodemographic information, reproductive history and gynecological surgery history.

Quality of Discharge Teaching Scale was developed by Weiss et al. [18] and consisted of 24 items, including 3 dimensions: Content needed (6 items), Content received (6 items), and Delivery (12 items). The Quality of Discharge Teaching Scale is a patient self-reported measure that uses an 11-point (0 to 10) scale. The total score is calculated by adding the scores of “Content received” and “Delivery”. The total possible score ranges from 0 to 180. Higher scores indicate a greater amount of discharge-related information received and a higher-quality teaching approach [33]. The “Delivery” dimension measures participants’ perception of the skills of doctors and nurses as health educators. In a previous study, the Cronbach’s α coefficient for the Quality of Discharge Teaching Scale was 0.92, and for the 3 dimensions, it ranged from 0.85 to 0.93 [18, 25]. The Cronbach’s α coefficient of the Chinese version was 0.91, and the range of the 3 dimensions was from 0.882 to 0.935. The Chinese version was authorized by the original authors [34].

The Readiness for Hospital Discharge Scale was developed by Weiss et al. [18] and consists of 22 items, including 4 dimensions: Physical condition (6 items), Knowledge (8 items), Coping ability (3 items), and Expected support (4 items). The Readiness for Hospital Discharge Scale is a self-reported scale with items scored on an 11-point scale (0–10). The total possible score ranges from 0 to 220. Higher scores indicate greater readiness. The Cronbach’s α coefficient for the Readiness for Hospital Discharge Scale was 0.93 [18]. The Cronbach’s α coefficient of the Chinese version was 0.97 [35] and 0.89, 0.92, 0.90, and 0.85 for the 4 dimensions [24]. The Chinese version was authorized by the original authors [35].

The postsurgical outcomes form is a self-designed tool to record problems on postoperative Day 1, postoperative Day 7, and postoperative Day 28. The postsurgical problems included surgical site infection, pain, body temperature, bowel dysfunction, urinary tract dysfunction and vaginal bleeding.

On the day of discharge, patients received the general information questionnaire, Quality of Discharge Teaching Scale, and Readiness for Hospital Discharge Scale. On postoperative Day 1, postoperative Day 7, and postoperative Day 28, patients received the postsurgical outcomes forms through a follow-up system on a smartphone app. The trained investigators checked the data and then telephoned and interviewed the patients who needed medical intervention. Readmissions and deaths were also recorded.

### Statistical analysis

SPSS 21.0 (SPSS Inc, Chicago, IL) was used for statistical analysis. Median, standardized median, interquartile range (IQR), number (N) and percentage (%) were used to describe the demographic and clinical variables. Spearman correlations were conducted to explore the correlation between the Quality of Discharge Teaching Scale and Readiness for Hospital Discharge Scale. Generalized estimating equations were used to explore the determinants of postsurgical outcomes. Odds ratios (ORs) were calculated in generalized estimating equations. In all analyses, a P value of < 0.05 indicated statistical significance.

### Ethics approval and consent to participate

Our study was approved by the ethics committee. After providing written informed consent, participants received the questionnaires.

## Results

### Demographic and clinical characteristics

A total of 168 gynecological patients who underwent day surgery were recruited. The participants’ demographic and clinical characteristics are shown in Table 1.

**Table 1:** Demographic and clinical characteristics of participants (N = 168)

Variable	Median (IQR)/n (%)
Age	30.0 (27.0, 33.0)
Race	
Han	157 (93.5)
Minority	11 (6.5)
Marital status	
Unmarried	15 (8.9)
Married	153 (91.1)
Education level	
High school or below	59 (35.1)
College or above	109 (64.9)
Residence	
Rural area	38 (22.6)
Urban area	130 (77.4)
Family per capita monthly income (yuan)	
< 5000	84 (50.0)
≥ 5000	84 (50.0)
Natural delivery history	
Yes	26 (15.5)
No	142 (84.5)
Cesarean delivery history	
Yes	24 (14.3)
No	144 (85.7)
History of gynecological surgery	
Yes	59 (35.1)
No	109 (64.9)

### Quality of discharge teaching and readiness for hospital discharge

The scores of the Quality of Discharge Teaching Scale and Readiness for Hospital Discharge Scale are shown in Table 2. Responses to the first item of the Readiness for Hospital Discharge Scale indicated that 98.8% of the participants stated they were ready for discharge.

**Table 2:** The Quality of Discharge Teaching Scale and Readiness for Hospital Discharge Scale scores

Dimension	Number of items	Median(IQR)	Item median (IQR)
Quality of Discharge Teaching Scale			
Content needed	6	60.00 (51.50, 60.00)	10.00 (8.50, 10.00)
Content received	6	59.50 (49.00, 60.00)	9.92 (8.17, 10.00)
Delivery	12	120.00 (108.00, 120.00)	10.00 (9.00, 10.00)
Total	18	176.50 (157.50, 180.00)	9.81 (8.46, 10.00)
Readiness for Hospital Discharge Scale			
Physical conditions	7	53.50 (46.00, 69.00)	7.64 (6.57, 9.86)
Knowledge	8	75.00 (66.00, 80.00)	9.38 (8.25, 10.00)
Coping ability	3	29.00 (24.25, 30.00)	9.67 (8.08, 10.00)
Social support	4	39.00 (32.00, 40.00)	9.75 (8.00, 10.00)
Total	22	194.00 (173.00, 214.50)	8.82 (7.86, 9.75)

### Postsurgical outcomes

No readmissions and no deaths were recorded on postoperative Day 1, postoperative Day 7 or postoperative Day 28. The incidence of postsurgical outcomes on postoperative Day 1, postoperative Day 7 and postoperative Day 28 are shown in Table 3.

**Table 3:** The incidence of postsurgical outcomes on postoperative day 1, postoperative day 7 and postoperative day 28 (N = 168)

	postoperative day 1	postoperative day 7	postoperative day 28
Surgical site infection	0 (0.0)	0 (0.0)	0 (0.0)
Mild pain	100 (59.5)	50 (29.8)	5 (3.0)
Moderate pain	5 (3.0)	1 (0.6)	0 (0.0)
Fever	1 (0.6)	0 (0.0)	0 (0.0)
Bowel dysfunction	4 (2.4)	3 (1.8)	0 (0.0)
Urinary tract dysfunction	5 (3.0)	1 (0.6)	0 (0.0)
Vaginal bleeding	123 (73.2)	98 (58.3)	0 (0.0)
No complications	27 (16.1)	65 (38.7)	163 (97.0)
1 complication	47 (28.0)	54 (32.1)	5 (3.0)
2 complications	80 (47.6)	46 (27.4)	0 (0.0)
3 complications	12 (7.1)	12 (7.1)	0 (0.0)
4 complications	2 (1.2)	1 (0.6)	0 (0.0)

### Correlation analysis between Quality of Discharge Teaching Scale and Readiness for Hospital Discharge Scale

Scores of “Content needed”, “Content received”, “Delivery” and total score of Quality of Discharge Teaching Scale showed strong positive correlations with scores on all dimensions of Readiness for Hospital Discharge Scale (P < 0.001). More details are shown in Table 4.

**Table 4:** Correlations between Quality of Discharge Teaching Scale and Readiness for Hospital Discharge Scale

Dimension	Physical conditions	Knowledge	Coping ability	Social support	Total score of Readiness for Hospital Discharge Scale
Content needed	0.354 (< 0.001)	0.625 (< 0.001)	0.499 (< 0.001)	0.534 (< 0.001)	0.525 (< 0.001)
Content received	0.402 (< 0.001)	0.662 (< 0.001)	0.521 (< 0.001)	0.558 (< 0.001)	0.573 (< 0.001)
Delivery	0.277 (< 0.001)	0.605 (< 0.001)	0.503 (< 0.001)	0.573 (< 0.001)	0.495 (< 0.001)
Total score of Quality of Discharge Teaching Scale	0.356 (< 0.001)	0.683 (< 0.001)	0.536(< 0.001)	0.600 (< 0.001)	0.571 (< 0.001)

### Generalized estimating equations of postsurgical outcomes

“Delivery”, “Physical conditions” and “Social support” of the Readiness for Hospital Discharge Scale were protective factors for postsurgical outcomes. More details are shown in Table 5.

**Table 5:** Generalized estimating equations of postsurgical outcomes

Independent variable	B	SE	Wald χ^2^	P	OR	OR 95% CI
Age	− 0.011	0.017	0.441	0.507	0.989	0.956, 1.022
Race = Han	− 0.214	0.320	0.447	0.504	0.807	0.431, 1.512
Education level = High school or below	0.023	0.151	0.023	0.879	1.023	0.761, 1.375
Marital status = Unmarried	− 0.481	0.247	3.775	0.052	0.618	0.381, 1.004
Residence = Rural area	0.264	0.178	2.203	0.138	1.302	0.919, 1.844
Family per capita monthly income (yuan) ≤ 5000	0.055	0.138	0.161	0.688	1.057	0.807, 1.384
Natural delivery history=No	0.402	0.234	2.953	0.086	1.498	0.945, 2.365
Cesarean delivery history=No	− 0.335	0.223	2.259	0.133	0.715	0.462, 1.107
History of gynecological surgery=No	0.023	0.164	0.020	0.889	1.023	0.742, 1.410
Content needed	0.032	0.016	4.057	0.083	1.029	0.996, 1.063
Content received	0.003	0.020	0.030	0.862	1.003	0.965, 1.044
Delivery	− 0.019	0.008	5.314	0.021*	0.981	0.965, 0.997
Physical conditions	− 0.036	0.008	22.451	< 0.001*	0.964	0.950, 0.979
Knowledge	− 0.009	0.012	0.575	0.448	0.991	0.967, 1.015
Coping ability	0.012	0.030	0.154	0.695	1.012	0.953, 1.074
Social support	− 0.047	0.022	4.446	0.035*	0.955	0.914, 0.997

## Discussion

### The status of Quality of Discharge Teaching Scale and Readiness for Hospital Discharge Scale

The item median score of the Quality of Discharge Teaching Scale for gynecological patients who underwent day surgery was 9.81, higher than previous studies on parents of hospitalized children, postpartum women, cataract patients and general surgical patients [14, 15, 22, 25, 36]. The score of “Content needed” was slightly higher than the score of “Content received”, but the difference in these two variables was not statistically significant (P > 0.05), which was inconsistent with previous studies of postpartum women and cataract patients [24, 25]. These results reflected that the content that participants received about discharge almost met their needs. Moreover, doctors and nurses provided good quality discharge teaching for these patients despite their decreased length of hospital stay. For the “Delivery” dimension, the item median score was relatively higher than previous studies [14, 15, 22, 25, 36], which indicated that the discharge teaching skills and effects of doctors and nurses were acceptable for gynecological patients who underwent day surgery.

Interestingly, for the Readiness for Hospital Discharge Scale, the item median score was 8.82, and 98.8% of the participants stated they were ready for discharge, which was higher than that in previous studies of other populations [24, 25, 36, 37]. One reason might be the relatively young age of our participants. As reported, the older patients were, the more likely they were to be not ready for discharge [38]. Another reason might be the preoperative assessments, which screened the patients’ physical condition, as mentioned above, the physical conditions is the dimension of the Readiness for Hospital Discharge Scale [18]. Moreover, day surgery was only recommended for patients with milder conditions and no comorbidities. Therefore, a variety of reasons caused this difference and we must acknowledge the differences. The score of “Social support” was rated highest, followed by “Coping ability” and “Knowledge”. The score of “Physical conditions” was rated lowest. We inferred that the reason might be the decreased length of hospital stay. Because gynecological patients who underwent day surgery were discharged in an intermediate stage of recovery, we recommend that doctors and nurses be more concerned about their physical condition, ensure their comfort and teach them how to manage postsurgical problems effectively.

### The correlation between the Quality of Discharge Teaching Scale and Readiness for Hospital Discharge Scale

As expected, the Quality of Discharge Teaching Scale was verified to be positively correlated with Readiness for Hospital Discharge Scale, and each dimension of the Quality of Discharge Teaching Scale and Readiness for Hospital Discharge Scale showed strong positive correlations. These results were consistent with previous studies on cataract patients, parents of hospitalized children, general surgical patients and psychiatric patients [22, 23, 36, 39]. The findings support the structure and relationships proposed by Meleis’ middle-range theory of transitions. Discharge teaching as a medical therapeutic process was verified to be a predictor of readiness for hospital discharge. As mentioned above, the decreased length of hospital stay of gynecological patients undergoing day surgery might make it challenging for doctors and nurses to deliver high-quality discharge teaching. Considering that patients’ readiness for hospital discharge was affected by the discharge teaching, we recommend that human resource managers ensure the number of doctors and nurses to avoid compromising the quality of discharge teaching due to workload. Future research could explore how doctors and nurses can effectively deliver discharge teaching during limited hospital stays.

### The determinants of postsurgical outcomes

Most patient-reported problems after minimally invasive gynecologic surgery include pain, vaginal bleeding, urinary tract infection and surgical site infection [26–28]. This study showed that the most common postsurgical problems of minimally invasive gynecologic surgery were vaginal bleeding, followed by mild pain. In the generalized estimating equations of postsurgical outcomes, “Delivery” of Quality of Discharge Teaching Scale, “Physical conditions” and “Social support” of Readiness for Hospital Discharge Scale were protective factors for postsurgical outcomes. No demographic or clinical characteristics of participants were statistically significant in generalized estimating equations. These findings were partly consistent with a previous study on cataract patients, in which “Content needed” and “Delivery” both affected the patient’s postsurgical outcomes through the mediating effect of Readiness for Hospital Discharge Scale [24]. These findings indicated that the higher the level of skills and effects of discharge teaching of doctors and nurses, the better the postsurgical outcomes in our patient population. Moreover, these findings support the structure and relationships of the theoretical framework based on Meleis’ middle-range theory of transitions. Discharge teaching as a medical therapeutic process was verified to be a predictor of postsurgical outcomes. Therefore, we recommend that human resource managers should train doctors and nurses in discharge teaching methods. Improving the health teaching skills and competencies of doctors and nurses is an important way for patients to improve postsurgical outcomes. Another prior study on patients diagnosed with anxiety disorders showed that a higher level of “Knowledge” of Readiness for Hospital Discharge Scale showed a lower risk of unscheduled clinic visits and readmission, and a higher level of “Social support” also showed a lower risk of unscheduled clinic visits. This study found that better physical conditions and a higher level of social support at hospital discharge were associated with better postsurgical outcomes. This finding suggests that doctors and nurses should assess patients’ physical conditions at discharge, including pain, strength and physical ability. Although doctors and nurses might not be able to change patients’ physical conditions through discharge teaching, they could teach them how to reduce stress and manage their pain via cognitive–behavioral therapy [39]. Moreover, social support, which is defined as informational, emotional, appraisal and practical support from partner, family or friends, should be valued [40, 41]. Previous studies also verified the positive relationship between social support and physical condition and quality of life [41–44]. Therefore, for gynecological patients undergoing day surgery, we recommend that in addition to clinical care, doctors and nurses take every opportunity to give appropriate and personalized information and appraisals and emotional and practical support. In addition, facilitating the patients’ social support system is crucial.

## Limitations

There are three limitations in this study. First, the median age of participants in this study was relatively young (30.0), so our findings may differ from those of older gynecological patients. Second, our participants underwent day surgery, so the findings might differ from those of patients hospitalized for surgery. Third, the sample may not be representative of all gynecological patients undergoing day surgery in China because participants were recruited from one tertiary hospital in western China.

## Conclusions

Considering that patient readiness for hospital discharge was affected by discharge teaching, we recommend that human resource managers ensure the number of medical staff to avoid compromising the quality of discharge teaching due to workload. Doctors and nurses should improve their discharge teaching skills and effects. Moreover, the findings indicated that the better the physical conditions and the higher the level of social support at hospital discharge were, the better the postsurgical outcomes. Doctors and nurses should assess patients’ physical conditions at discharge and teach them how to reduce stress and manage postsurgical problems. Facilitating the patients’ social support system is also crucial.

## Data Availability

The data that support the findings of this study are available from West China Second University Hospital but restrictions apply to the availability of these data, which were used under license for the current study, and so are not publicly available. Data are however available from the authors upon reasonable request and with permission of West China Second University Hospital.
